# Development of a Nonwoven Hemostatic Dressing Based on Unbleached Cotton: A De Novo Design Approach

**DOI:** 10.3390/pharmaceutics12070609

**Published:** 2020-06-30

**Authors:** J. Vincent Edwards, Elena Graves, Nicolette Prevost, Brian Condon, Dorne Yager, Joseph Dacorta, Alvin Bopp

**Affiliations:** 1 Southern Regional Research Center, New Orleans, LA 70124, USA; elena.graves@usda.gov (E.G.); nicolette.prevost@usda.gov (N.P.); brian.condon@usda.gov (B.C.); 2 Plastic and Reconstructive Surgery, Virginia Commonwealth University, Richmond, VA 23111, USA; dorne.yager@vcuhealth.org; 3 H&H Medical Corporation, Williamsburg, VA 23185, USA; jdacorta@gohandh.com; 4 Department of Natural Sciences, Southern University at New Orleans, New Orleans, LA 70126, USA; ABopp@suno.edu

**Keywords:** cotton, blood clotting, thromboelastography, nonwovens, electrokinetic, fibrin, thrombin, platelets

## Abstract

Minimally processed greige (unbleached) cotton fibers demonstrate enhanced clotting relative to highly processed *United States Pharmacopeia* (USP) type 7 bleached cotton gauze. This effect is thought to be due to the material surface polarity. We hypothesized that a textile could be constructed, conserving the hemostasis-accelerating properties of greige cotton, while maintaining structural integrity and improving absorbance. Spun bond nonwovens of varying surface polarity were designed and prepared based on ratios of greige cotton/bleached cotton/polypropylene fibers. A thromboelastographic analysis was performed on fibrous samples in citrated blood to evaluate the rate of fibrin and clot formation. Lee White clotting times were obtained to assess the material’s clotting activity in platelet fresh blood. An electrokinetic analysis of samples was performed to analyze for material surface polarity. Hemostatic properties varied with composition ratios, fiber density, and fabric fenestration. The determinations of the surface polarity of cotton fabrics with electrokinetic analysis uncovered a range of surface polarities implicated in fabric-initiated clotting; a three-point design approach was employed with the combined use of thromboelastography, thrombin velocity index, Lee White clotting, and absorption capacity determinations applied to fabric structure versus function analysis. The resulting analysis demonstrates that greige cotton may be utilized, along with hydrophilic and hydrophobic fibers, to improve the initiation of fibrin formation and a decrease in clotting time in hemostatic dressings suitable to be commercially developed. Hydroentanglement is an efficient and effective process for imparting structural integrity to cotton-based textiles, while conserving hemostatic function.

## 1. Introduction

**Figure 1:**
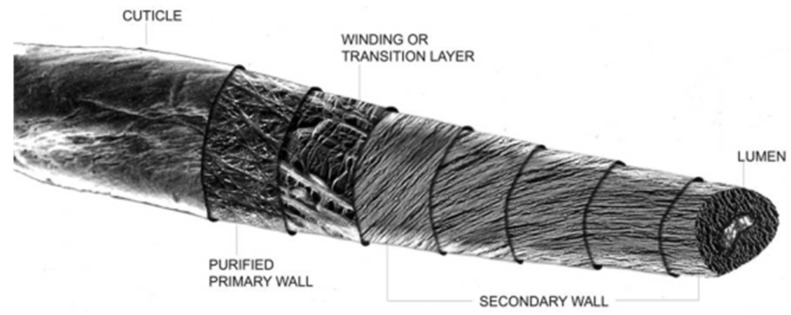
A montage of cotton fiber sections constructed from individual electron micrographs (TEMs) and scanning micrographs (SEMs) of the fiber. Gives an overview of the layered structures of the fiber. The fiber cuticle, primary and secondary cell walls have been shown at different magnifications, to better visualize fibrillary structures and fiber layers from the surface to the lumen [3].

Cotton has been a dressing staple for wound tamponade establishment for centuries. With the exception of its use in historically high demand periods, as in the ancient times of battle and the American Civil War, greige unbleached cotton has been relatively unexplored with regard to hemostatic dressing applications [1,2]. Nonwoven materials utilizing traditional fibers, such as bleached cotton, rayon and polyester, are utilized in hemostatic gauze. Due to innovations in cleaning and hydroentanglement, there is growing interest in the potential use of greige cotton in nonwoven dressings [3,4]. As shown in Figure 1, the cotton fiber consists of an outer protective cuticle, containing hydrophobic lipids that are only a few percent by the weight of the total fiber [3]. Nonwoven unbleached cotton retains the cuticle lipids, which confer a hydrophobic character presented as a thin waxy layer across the fiber surface. The lipid-containing layer is loosened from the cellulosic primary cell wall during the hydroentanglement process, and thus, exposes the cellulosic secondary cell wall. It is the hydrophilic cellulosic portion of the cotton fibers that confers wettability and absorbency. The combination of a hydrophobic and hydrophilic character (termed amphiphilicity) in hydroentangled greige cotton materials confers a fluid management property that has functionality as a skin-contacting material, as is found with hygienic top sheets and wound dressings [4,5]. These same properties may make greige cotton ideally suited as a hemostatic material for dressing development.

Most of the current literature tends to characterize hemostatic textile-based materials as addressing hemorrhage control. However, a broader measure of hemostatic material types applies to dressings that accelerate surface hemostasis, and categorically are either untreated or treated textile or granular materials [6,7,8]. Treated dressings are typically woven or nonwoven textile materials that have a hemostatic agent incorporated, i.e., clay minerals, chitosan (used singularly as a fiber or coating), modified polysaccharides and/or fibrin sealant as the active clotting agent [6]. Moreover, these types of dressings, which contain hemostasis-activating agents, have been classified as either factor concentrators, pro-coagulants, or mucoadhesives, based on their mechanism of action to initiate and sustain blood coagulation [7,8]. On the other hand, dressings which demonstrate a degree of hemostatic activity based on de novo design of woven or nonwoven textiles have been developed from single or multiple fiber blends [9]. In this case, the structure/function relationship of fiber and material design often varies, based on surface area, charge, polarity, surface morphology, the material’s porosity, wicking function and absorption capacity, which are related directly to in vivo models, i.e., assessments done animal hemostasis models.

Despite the large number of dressings used for hemostasis, few reports have comprehensively evaluated the effect of material properties as separate and causative effects between and within interrelated fiber and fabric structural features. Approaches to evaluating the structure/function relations of materials for hemostatic performance have varied. Two contrasting perspectives in this regard are as follows: (1) focus on in vivo performance, versus (2) testing structure/function concepts with in vitro assays that determine the role of the coagulation cascade in initiation of clotting [9,10].

Approaches to assessing the role of coagulation cascade factors, fibrin formation and platelet adherence [11,12] have, for the last two decades, brought hemostatic dressings under investigation for their clotting activity, by applying the knowledge of blood platelet and coagulation cascade contact activation to material design [13,14]. The improvement of the hemostatic activity of time-honored dressings has also been a focus of this type of research [14]. Notably, dressing development has largely been based on naturally occurring fibers, such as cotton, chitosan, alginate, collagen and other polysaccharides and collagen, and it has led to a new generation of products and prototypes [12,15,16,17].

Research on hemostatic dressings with cellulose has also received increased attention in recent years. For example, reports on carboxymethylcellulose [18], oxidized and regenerated cellulose [19,20], and regioselectively modified cellulose [21], and unique forms of cellulose fibers [22,23] exemplify a resurgent interest in improving the hemostatic activities of naturally occurring materials. In this study, the use of the unbleached cotton fiber allows a new perspective on the cotton fiber’s secondary cell wall, which contains most of the cellulose cotton, accompanied by its intact primary cell wall and cuticle waxes. Developing a concept of the relation of surface chemistry to the induction of clotting will also facilitate the discovery and development of hemostatic fabrics.

### Triad Approach to Hemostatic Dressing Discovery

Approach designs to develop hemostatic dressings, as well as to further characterize the activity of long-established dressing products, have optimized the activity of dressings that promote and sustain coagulation. Traditionally, empirical approaches for measuring clotting to improve material functionality have been utilized, with the simplest approaches being to measure the wettability, absorption capacity and wicking activity of the absorbent material, and then relating that activity to the dressing’s effect in an in vivo model, as an indication of its functional performance to establish an effective tamponade to bleeding [19].

In recent years, there has been increasing interest in developing hemostatic dressings, based on an a priori understanding of the role of the dressing’s interaction with the clotting components of blood at a molecular level, by way of the coagulation cascade and platelet adhesion, with the dressing material’s contact surface properties [12]. Chitosan and alginate and constituent carbohydrate derivatives are examples of naturally occurring biopolymers using this approach [11,15,24,25]. Thus, approaches to develop hemostatic dressings, as well as to more fully characterize the activity of long established dressing products, have led to renewed interest in optimizing dressings that promote and sustain coagulation.

**Figure 2:**
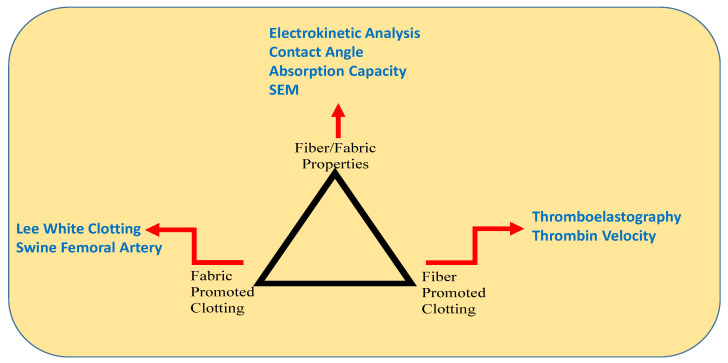
A diagram of the three-point approach taken, where fiber promoted clotting (as measured by thromboelastography (TEG)) were correlated with fabric and fiber surface polarity and absorption properties, as measured with electrokinetic profiles and absorption capacity, related to fabric promoted clotting, as measured with the Lee White clotting and thrombin assays.

We have utilized a triad approach (as diagrammed in Figure 2) to de novo dressing design and testing, that employs the analysis of coagulant activity at a fiber and fabric level. This approach is undertaken with a view to understanding the influence of material surface polarity on coagulant activity, in light of the functional properties of unbleached cotton. Here, we demonstrate how unbleached (greige) cotton, when blended with traditional nonwoven fibers to modulate fabric surface polarity, has an effect on initiating and promoting clot formation as a function of integrated compositional, polar surface, and fabric structural features. Furthermore, the optimization of clotting performance may be elicited from the structural properties of both the fiber and the nonwoven dressing design. This is done using an approach that employs electrokinetic and thromboelastography (TEG)-based structure function relations of fiber/fabric component discovery, coupled to absorption capacity and platelet-fresh in vitro clotting profiles of larger sections of the fabric material. Here, we illustrate how this approach can be applied to a stepwise development of a hemostatic dressing based on greige cotton.

## 2. Materials and Methods

### 2.1. Hydroentanglement of Fibrous Webs into Nonwoven Fabric Structure

Greige cotton fibers (True Cotton™) were obtained from T. J. Beall of Greenwood, Mississippi. These cotton fibers were pre-cleaned using a proprietary process, which removed 99.99% of all impurities (field trash, stems, leaves, etc.) in a nonaqueous, mechanical process (http://www.tjbeall.com/naturalfibers-nonwoven/true-cotton). Bleached cotton fibers were produced from a bale of True Cotton™ that were bleached by Tintoria Piana US in Cartersville, GA, USA. The polypropylene fibers were purchased from Fibervisions^®^ of Duluth, GA, USA. All fibers were received and used without further modifications. All nonwoven fabrics were prepared at the USDA Southern Regional Research Center in New Orleans, LA, USA. The manufacturing specifics are as follows: staple fibers were weighed and hand-blended, and then opened on an opening and blending line, consisting of a fiber hopper, Hollingsworth WR cleaner, D106 distributor, and 310 fine opener and another D106 distributor. All fiber blends were processed twice to achieve optimal openness and blending. The fiber blends were chute fed to a 40-inch-wide textile card fitted with four Cardmaster plates (Saco Lowell, Easley, SC, USA), followed by feeding the card web into a commercial crosslapper and needlepunch (NP) machine (TECHNOplants s.r.l., Pistoia, Italy). The NP processing parameters were varied to achieve the target weights (25–35 g/sqm). The NP fabrics were converted into hydroentangled (HE) nonwoven fabrics on a one-meter-wide Fleissner pilot-scale hydroentanglement system (Trützschler Nonwovens GmbH, Dülmen, Germany), running at a constant production speed of 5 m/min. The hydroentanglement (HE) system utilized two pressure heads: one low pressure for fabric wet-out maintained at a constant pressure of 30 bar during fabric production; and the second is a high-pressure head maintained at 60, 80, and 100 bar during separate fabric production runs. The final bonding nozzle is suspended over a 24-mesh embossing drum to cast a 24-mesh pattern into the nonwoven material. Each strip on the bonding pressure heads consisted of 50 orifices per inch, with an orifice pore size of 120 µm. The water used for the HE fabric production was at an ambient temperature, which was approximately 25 °C. Following HE, the fabrics were fed directly through a gas-fired fabric-drying oven (Trützschler Nonwovens GmbH) at ~160–180 °C (depending on fiber melt points) and wound into rolls.

### 2.2. Thromboelastography

Thromboelastography (TEG) assesses the viscoelastic properties of whole blood under low shear conditions and provides information about global hemostatic function from the beginning of clot formation through clot retraction and fibrinolysis [26]. Thromboelastography was performed at 37 °C on a TEG 5000 Thrombelastograph^®^ Hemostasis Analyzer System, using the TEG analytical software 4.2.3 (Haemonetics Corporation, Niles, IL, USA). Twenty microliters of citrated saline, 5.375 mM disodium citrate, 146 mM NaCl was added to the cup in each channel (2) containing fabric sample, 1 mg. To this, 30 µL calcium chloride (0.2 M) and 310 µL citrated bovine blood were added and the analysis was started immediately. Multiple runs were performed on each sample and subject to an Excel statistical analysis using the descriptive analysis. The resulting values were plotted versus greige cotton, bleached cotton, and polypropylene percent compositions in an Insight^TM^ program on *x*, *y*, *z* axes, with fabric density and time to fibrin formation integrated in one graph. The visualization of the time range to fibrin formation was completed with an Insight-based color spectrum program. 

### 2.3. Modified Lee White Clotting Assay

Freshly obtained swine blood (330 mL) was obtained by direct transfer from the animal to 35 mL of citrate phosphate dextrose adenine (CPDA-1). Each fabric sample was weighed (4–5 milligrams) and transferred to a test tube. Samples were run at least in triplicate and 2.5 mL of blood was added to each tube containing the fabric sample. To test the effect of the fabric on whole blood clotting, a calcium chloride solution was added to the sample tubes within a 45–60 s interval. Tubes were inverted upon addition of CaCl_2_ and placed at 37 °C. Visual assessment to clot formation was assessed by taking measurements at 1-min intervals. Multiple samples and runs were done, and a statistical analysis was completed using all Pairwise Multiple Comparison Procedures (Holm–Sidak method): overall significance level = 0.05.

### 2.4. Thrombin Assay 

Thrombin generation was assayed using a modified fluorogenic method, Technothrombin TGA (Technoclone, Vienna, Austria). Approximately 10 mg fabric was added to the sample well of a 24-well microplate, with 200 µL TGA buffer (hepes-NaCl-buffer containing 0.5% bovine serum albumin). To this were added 300 µL TGA reagent D (phospholipid micelles in Tris-Hepes-NaCl buffer), 300 µL citrated bovine plasma (Quad Five, Ryegate, Montana, Canada) and 500 µL of the calcium-fluorogenic substrate (1 mM substrate; 15 mM CaCl_2_). Fluorescence readings were started immediately after addition of the substrate and were read for 60 min, in 1 min measurement intervals. Readings were made on a BioTek Synergy HT reader (BioTek Instruments, Winooski, VT, USA) at 37 °C. Multiple runs were done and a statistical analysis was completed using all pairwise multiple comparison procedures (Holm–Sidak method): overall significance level = 0.05.

### 2.5. Zeta Potential Measurements

The determination of the *ζ*-potential was carried out with the SurPASS™ Electrokinetic Analyzer (Anton Paar, Ashland, VA, USA) using the Cylindrical Cell developed for the measurement of fiber and powder samples. When a fiber absorbs liquid and swells, the surface charges become farther separated, and the absolute value of its *ζ*-potential decreases. Two kinds of measurements were made on each sample: (1) swell tests to measure the rate and extent of fiber swelling (at a given pH) and (2) a pH titration, in which the swelling is measured as a function of pH. All *ζ*-potential measurements were made in a 1 mM KCl electrolyte. In the electrokinetic apparatus, the streaming potential is measured, and the zeta potential is determined from the Helmholtz–Smoluchowski equation,

(1) $$\zeta =\frac{dU}{dp}\times \frac{\eta }{\varepsilon \varepsilon_{0}}\times \kappa$$

where *U* is the streaming potential—the potential generated when an electrolyte is forced to flow over a stationary charged surface, *p* the pressure, *η* is the viscosity of the electrolyte solution, *ε* is the dielectric constant of the electrolyte, *ε*_0_ is the vacuum permittivity and *κ* is the electrolyte conductivity. The surface conductivity of the fibrous samples was not taken into account. pH titrations were performed over a pH range of 11.0 to 2.5, to ensure the recording of both the isoelectric point (IEP) and the plateau potential. The IEP is the pH at which zeta potential = zero, and provides insights into the surface association/dissociation processes. The swell test is done by applying a Mathcad program to the electrokinetic data. A swell test measures the rate and extent of fiber swelling. Swelling follows first order rate kinetics (Equation (2)), which, upon integration, gives Equation (3). Thus, these relationships are provided by way of the parameters of initial zeta (*ζ*_0_), final zeta (*ζ*_∞_) potential and rate of swelling (*k*). They also define a measure of water uptake ability and are used to derive the swell ratio as a measure of fiber swelling as well.

(2) $$\frac{d\zeta }{dt}=-k\left(\zeta_{0}-\zeta_{\infty }\right)$$

(3) $$\ln\left\{\frac{\left(\zeta -\zeta_{\infty }\right)}{\left(\zeta_{0}-\zeta_{\infty }\right)}\right\}=-kt$$

Three parameters (*ζ*_0_, *ζ*_∞_ and *k*) provide information on the fiber: (1) Δ*ζ* (change in *ζ* normalized to *ζ*_0_) = (*ζ*_0_ − *ζ*_∞_)/*ζ*_0_; water uptake ability; (2) swell ratio (measure of fiber swelling) = (*ζ*_0_/*ζ*_∞_)^1/2^; and (3) rate constant for fiber swelling.

### 2.6. Absorbency

A sample, consisting of 4 coupons, is measured and weighed, so that a “density” in g/cm^2^ can be determined. The coupons are soaked in a 142 mM NaCl/2.5 mM CaCl_2_ solution, removed on to a porous surface to drain the unbound solution for five minutes, weighed, centrifuged in a tube containing a wire mesh placed at the bottom of the tube to separate the centrifuged water, and reweighed. The mass difference of bound (intra-fiber water retained by fibers) versus inter-fiber bound water (weight of coupons minus the water removed) allows for the determination of the quantities of water that can be taken up and held at an inter-fiber (water held between the fibers of the fabric) and intra-fiber (water retained within the fibers) level. Although absorbency typically has been reported as g H_2_O/g fabric, with the density value, the absorbency in g/100 cm^2^ can also be calculated. Multiple runs were performed and the statistical analysis was completed with ANOVA, *p* = 0.05.

## 3. Results

### 3.1. Identification of Hemostatic Greige Cotton Nonwovens

Table 1 shows that the time to fibrin and clot formation observed in 100% greige cotton nonwoven is significantly shorter than with a bleached 100% cotton sample; *p* ≤ 0.05. The tendency for greige cotton to promote accelerated clotting is supported by prior work, where we demonstrated fabric-promoted clotting by way of incorporating unbleached cotton with combinations of hydrophilic and hydrophobic fibers, i.e., clotting times correlated to changes in material surface polarity [4]. In addition, the TEG-determined clotting times (*k* values) for the fabrics discussed in that study correlated with thrombin velocity, as measured independently with a thrombin fluorescent substrate [4]. Thus, the previous study demonstrated the utility of using a TEG-based structure/function approach to assist in the design of cotton-based hemostatic fabrics. Here, we show how this approach, as outlined in Figure 2, is used in the design and development of a dressing with improved bleeding control properties.

The triad approach for hemostatic dressing design, Figure 2, is utilized to develop a structure/function relation, with fiber combinations that confer hydrophilic and hydrophobic character to the dressing fabric. Thus, the underlying concept is that the modulation of surface polarity by way of incorporating hydrophilic and hydrophobic fibers confers clotting properties to the blood-contacting surface, and influences the initiation of the clotting cascade [27].

### 3.2. Examination of Fabric Composition, Mesh and Density with a Model Fabric 

The effectiveness of using thromboelastography (TEG) to assess the hemostatic activity of biomaterials has been demonstrated in a variety of materials, including films, colloids, and glass-like fibers [4,26]. Here, we have employed TEG to examine the effect of fiber composition, mesh, and density on clotting performance. These properties influence surface area and polarity and mediate absorption capacity. However, despite the large body of literature on hemostatic materials, reports on the examination of the inter-related effect of fabric and fiber-promoted clotting are scarce. 

**Figure 3:**
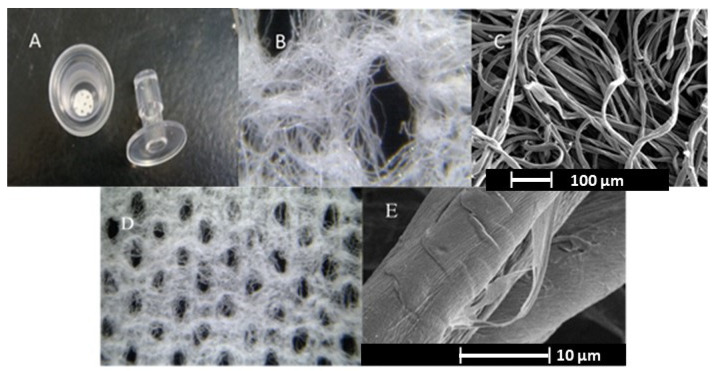
The images shown include: (**A**) nonwoven fabric placed in a thromboelastography cup, (**B**) hydroentangled greige cotton magnification 130×; (**C**) SEM of greige cotton fibers. Note cracks in the cuticle where pectin and absorbent cellulose are exposed and; (**D**) hydroentangled greige cotton magnification 26×; (**E**) SEM of greige cotton fiber taken from a nonwoven showing cuticle lifted above the surface from pressure of waterjets during HE process.

To help visualize the relation of fiber to fabric structure, a set of fabric and fiber images at different magnifications is shown in Figure 3. The image of a cross section of fabric sample in a thromboelastography cup is shown in Figure 3A, and images of the fabric structure (D) are shown adjacent to images of the fibrous web at two magnifications (Figure 3B,C). Image E is an SEM of the disposition of cotton fibers and loosened cuticle surface from nonwoven hydroentanglement. The contrast of these features of the fabric and fiber at different levels of magnification is helpful to demonstrate that milligram amounts of nonwoven fibers (as shown in the TEG sample cup, Figure 3) are a representative cross-section of the material’s surface upon contact with blood. Each thromboelastography experiment is done with a fabric/fiber disc that is a centimeter in diameter (there are roughly fifteen hundred to three thousand fibers assembled within the surface and shape dimensions of the fabric disc placed in the thromboelastography cup).

**Figure 4:**
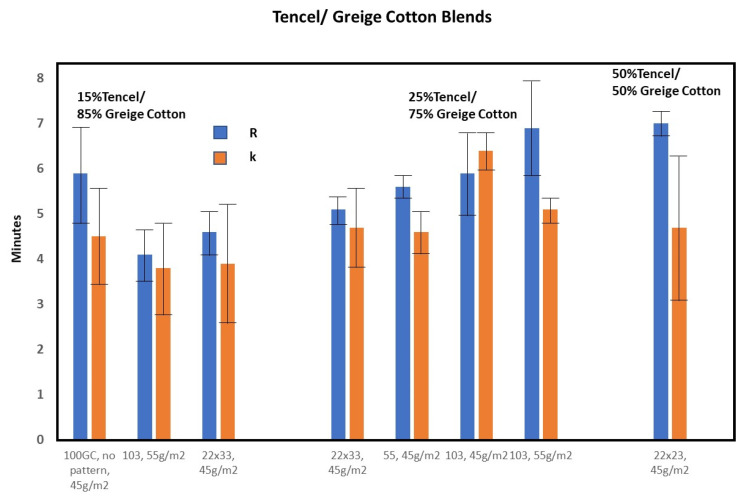
A comparison of thromboelastography (*R* = fibrin formation = blue, *k* = clot formation = orange) results for three nonwoven compositions, wherein density and mesh were varied. SD = standard deviation, *n* = 3.

To measure the effect of varying fabric composition, the mesh and density on TEG profiled clotting a tightly controlled set of fabric structures were obtained from a commercial nonwovens producer. In this process, Tencel and greige cotton fibers were combined in commercial hydroentangled nonwoven fabrics. Tencel is a form of lyocell, a rayon-like cellulose fiber taken from bleached wood pulp [28]. For comparison, fabrics with a fenestrated pattern prepared with three different mesh screens (103, 55 and 22/23 at two fabric densities), during the HE process, were compared with a non-fenestrated fabric. Note the fabric fenestration results in the pattern depicted in Figure 3B,D. The results of the fabric’s structural effects on TEG-determined fibrin formation are shown in Figure 4. There was considerable overlap in time to fibrin formation (R) and there was no significant difference in the rate of fibrin formation for this set of fabrics tested; *p* > 0.05. However, shorter time to clotting (*k*) is evident in the fabric composition 85% greige cotton/15% Tencel, with a density of 45 g/m^2^ at both 103 or 23 mesh values when compared to non-fenestrated fabric. In the 75% greige cotton/15% Tencel composition, thromboelastography profiles were indicative of a shorter time to clot formation with the 45 g/m^2^ density fabric; 22/23 mesh compared with 55 and 103 mesh values.

### 3.3. Construction of Cotton-Based Nonwovens for Clotting Structure/Function Relations 

**Figure 5:**
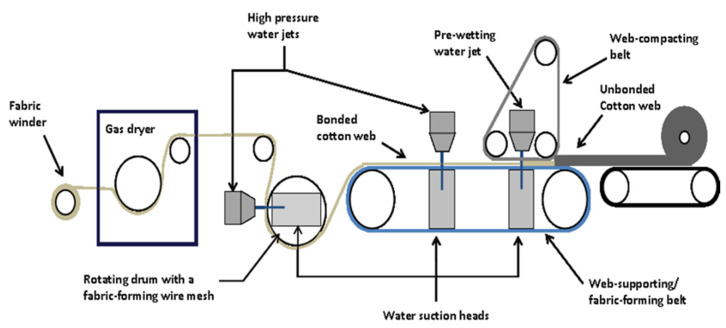
Diagram of a nonwoven hydroentanglement line. An outline schematic of the Fleissner MiniJet system used in the study.

The modeling study employing greige cotton/Tencel blends was undertaken, to determine the type of mesh screen to impart fenestration during nonwovens production of the cotton-based dressing. The results of the study discussed above suggest a 22/23 mesh screen be utilized in the construction of hydroentangled fabrics. Thus, in the next phase of development, a series of ten fabrics were prepared on a pilot mill hydroentanglement line, diagrammed in Figure 5. Fabrics were prepared in fiber ratios designed to vary the material surface polarity and to examine structure/function relations using the triad approach (Figure 2).

#### Modeling Fibrin Formation Based on Fabric Composition and Density

**Figure 6:**
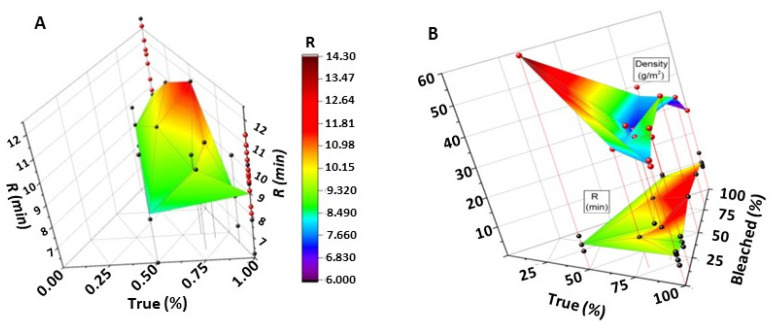
(**A**) Three-dimensional graphical methods that were employed to identify trends in clotting in terms of the three-component composition are shown. Thus, variation in blood clotting properties due to compositions (i.e., of greige cotton, bleached cotton, and polypropylene) in hydroentangled non-woven fabrics are visualized in 3D. Colored points represent actual sample determinations. The three bottom axes are greige cotton (True Cotton™) (front axis), bleached Cotton (right axis), and polypropylene (rear axis), and the vertical axis indicates R (the time to the onset of clot formation as measured by initial fibrin formation). (**B**) The 3D graphical methods that were employed to identify trends in absorbency are shown, that correlate to fabric density (as shown here) and blood clotting properties by varying compositions (i.e., of greige cotton, bleached cotton, and polypropylene) in hydroentangled non-woven fabrics. The three bottom axes are True Cotton™ (front axis), bleached Cotton (right axis), and polypropylene (rear axis). The left back axis is multi-unit (0–20 is minutes, and 20–60 g per square meter). The lower plate of data shown with a plateau at an *R* value of 10 min represents the effect of composition on *R* (min). Whereas the upper plate peaking at 60 gsm represents the relation of fabric composition and density to *R* (color-coded by time in minutes to clot formation, as shown in (**A**).

An assessment of the effect of fabric composition and density on the rate of fibrin formation was undertaken. The results of the structure function relation are shown in Figure 6. The plot was done with an Insight program, modeling the data in three dimensions. *R* values (time to fibrin formation) were plotted for fabrics hydroentangled at 60 bar (waterjet pressure of HE line). In Figure 6A, the compositions favoring greige cotton as a surface component to accelerated fibrin formation are on the lower left portion of the plot—the green areas nearer the *x*-axis are for greige cotton (labeled TC). Recall *R* is the time to the onset of clot formation by way of initiation of fibrin formation, so low values of *R* are desired to enhance accelerated surface contact activation of the coagulation cascade. Thus, the results of this analysis confirmed the importance of greige cotton within a larger data set as playing an important role in the early stages of clotting.

As shown in the upper panel of Figure 6B, the plots of time to fibrin formation vs. the density of the fabric compositions showed an inverse relation between density and time to fibrin formation i.e., fibers from lower density fabrics result in shorter fibrin formation times (*R*). This suggests that fabric void spaces or pores play a role in accelerating coagulation by way of surface contact activation [29], consistent with enhanced permeability and increased surface area [30,31]. This appears to be the case for lower density (less than 20 gsm) fabrics, as is evident in the upper data set of Figure 6B portrayed in the parabolic quadrangle. This result is also consistent with a negatively charged hydrophilic surface inducing contact activation [32]. On the other hand, the data set does not take into account the time to actual clot formation, which is also dependent on the increased wettability of dense hydrophilic fibers.

### 3.4. Electrokinetic Analysis 

The same ten compositional nonwoven blends were subjected to an electrokinetic analysis. It is important to note that the surface charge and polarity of some hemostatic fabrics and clay minerals have been shown to correlate to the initiation of blood clotting. An electrokinetic analysis allows for the analysis of material swelling. Zeta potential is an electrokinetic measurement at the aqueous shear plane (the boundary between a compact layer of ions and the diffuse layer), that is defined by the difference in the potentials between the shear plane and the electroneutral region. Zeta potential decrease is caused by the swelling of fiber/fabric properties and the corresponding outward movement of the aqueous shear plane ions, in contact with the outer Helmholtz plane (positive ions on the fiber surface) [33]. Fiber swelling results in an exchange of ions across the electrochemical double layer. In this way, the surface charges become farther separated and the absolute value of its *ζ*-potential decreases. Zeta potential titrations under these conditions give rise to a determination of the relative material polarity.

The results of the electrokinetic pH titrations of the fabrics are shown in Table 2. These were obtained to measure the relative surface polarities and compare the swell behavior of the fabrics to their effect on clotting. Zeta plateau (*ζ*_plateau_) is the functional value of material surface polarity and is taken from the zeta potential titration curve for the fabric. The zeta plateau (*ζ*_plateau_) for the fabrics of this study varied from −48 to −27. Thus, a 21 mV difference was observed in the *ζ*_plateau_ value of the fabrics evaluated, and the difference is associated with increasing hydrophobic fiber content. When evaluated for time to fibrin formation as compared with untreated blood controls, the polarity difference corresponded to a 30–40 percent decrease in time fibrin formation. Thus, although there is not a strict correlation between fibrin formation and electrokinetic properties as previously noted [4], the samples of the study align with a trend of increasing hydrophobicity, corresponding to decreasing time to fibrin formation; *p* ≤ 0.05. This result is consistent with the literature on the role of negatively charged hydrophobic surfaces initiating the clotting cascades through the binding of Factor XII to negatively charged surfaces [34].

Fiber swelling is identified in the electrokinetic analysis by delta zeta (Δ*ζ*). Fiber swelling is a function of the structure, the degree of crystallinity, and the amorphous and void regions of a fiber. In a fibrous web, the inter-fiber orientation of the web, the void spaces, and the surface polarity will affect the swelling of the fabric [35]. Fiber porosity influences the rate of fluid uptake, based on the thickness per unit mass, linear density, and density of the constituent fibers [29]. Thus, the relationship between swelling, as measured by Δ*ζ* and time to fibrin formation, have numerous determinant properties. The observed values of Δ*ζ* ranged from 0.14 to 0.05, and the corresponding percentage difference in decreased time to fibrin formation was 33 percent. Notably, fabric B1 (85/15, greige cotton/bleached cotton) with the highest swelling value also showed a relatively greater decrease in time to fibrin formation. The composition of B1 is also consistent with the results in Figure 6A, where higher ratios of greige cotton gave shorter times to fibrin formation. It is interesting that the composition of B1 is near that reported by Sperling et al., as an optimal mix of hydrophilic and hydrophobic surface polarity to initiate contact activation [27], albeit they are different types of surfaces. In this regard, it is important to note the distinction made in the literature between the effect of hydrophilic versus hydrophobic surfaces and the adsorption of key plasmatic proteins that influence coagulation on idealized monolayer surfaces [32]. Sperling et al. found significantly more activation with a mix of hydrophilic and hydrophobic molecular derivatization of monolayer surfaces [27]. In that study, the amount of hydrophobic surface (CH3 groups) was 17% and hydrophilic (COOH) 83% or mixed polar surfaces. Strong platelet adhesion was also found on hydrophobic surfaces when compared with a completely hydrophilic surface (100% COOH). With these types of surfaces, it was shown that a minimum of negative charge or distance between charged groups is also responsible for activation. 

### 3.5. Absorption Capacity

In Table 3, the absorption capacities of the nonwoven blends described are shown for both the inter- and intra-fiber absorption of physiological saline. The intra-fiber absorption capacity is the amount of water retained inside the fiber in addition to tightly bound fiber surface adherent water layers, and it is expected to be a considerably lower value as per the fiber volume than the inter-fiber absorption values for bound water. The intra-fiber absorption capacity values for the nonwovens of this study varied from 0.13–0.34 g/g. On the other hand, the bound inter-fiber water is water adhering between the fabric fibers and is retained by a combination of hydrogen bonding and van der Walls forces of water molecules between the fibers. Inter-fiber bound water varied from 1.84–17.54 g/g. Fabric B2 (greige cotton/bleached cotton/polypropylene, 30/50/20) had the highest absorption capacity, and one hundred percent nonwoven bleached cotton fiber (B7) was also within the standard deviation range of absorption capacity for B2; *p* > 0.05. This is interesting, since standard commercially bleached and scoured cotton gauze had sixty percent less absorption capacity, as measured by the inter-fiber absorption capacity. Moreover, the intra-fiber absorption values of commercially bleached and scoured cotton were ten times less than that found for B2 and B1. Commercially bleached and scoured cotton woven fabrics are stripped of both the cotton fiber cuticle and most of the primary cell wall. Thus, the outer cotton fiber structure is removed through the bleaching and scouring processes, leaving the crystalline secondary cell wall, which is principally cellulose, and although absorbent, has limited retention capacity. The results of the inter- and intra- fiber values suggest that water retention mediated by greige cotton fibers promotes higher absorption capacity. Notably, this is relevant in bleeding control, since the goal of pressure packing with traumatic wounds is to establish tamponade based on the cessation of blood flow. Moreover, the mechanism of rapid acquisition and retention by a dressing promotes coagulation. Interestingly, the combination of hydrophilic fibers with hydrophobic fibers as polypropylene and polyester has been found to work synergistically in a fibrous web to confer water distribution and retention properties [36]. This property (acquisition/retention) was demonstrated previously in the design of dressings and hygienic nonwovens [4,5,36,37]. 

### 3.6. Lee Clotting and Thrombin Generation Analysis

The final development step of the nonwoven cotton fabric as a hemostatic dressing involved selecting a lead based on the triad design approach (Figure 2). As shown above, the zeta potential reflects hydrophobicity. However, the degree of hydrophobicity can also be identified with contact angle measurements. The hydrophobicity of the cotton fiber is conferred by cuticle lipids which adhere to the outer surface (Figure 1). The cotton fiber cuticle lipids consist of a variety of organic functional groups, most of which are primary alcohols, but also included are fatty acids, aldehydes and alkanes, that vary in length from C16 to C36, yet make up less than one percent of the weight of greige cotton [38]. However, lipids from hydroentangled greige cotton are loosened, removed, and distributed to some extent on the surface from the waterjet pressure. Contact angles have been used to estimate hydrophobicity conferred by the lipids in nonwoven cotton, and the confirmation of the relation of contact angles to the samples of this work was estimated based on previous studies, with similar fabrics corresponding to a range from 90° to 120°, indicative of hydrophobicity [4,5].

**Figure 7:**
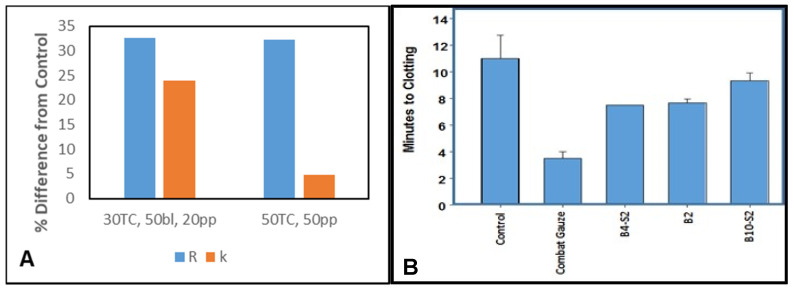
(**A**) Plots of the percent difference in R (time to fibrin formation) and *k* (time to clot formation), determined with thromboelastography between untreated blood control and fabric treated blood. (**B**) Lee White clotting assay results for (30/50/20, gc/bl/pp, B4-S2 and B2) and (33/33/33, gc/bl/pp, B10-S2), and a positive control containing a procoagulant added to the fabric. A plot of percent differences in R (time to fibrin formation) and *k* (time to clot formation) for nonwoven samples at a blend ratio of (30/50/20, gc/bl/pp) for three sets of fabrics produced at increasing hydroentanglement waterjet pressures (60, 80, and 100 bar). Standard deviations, *n* = 3, Statistical analysis done in Sigma Plot with All Pairwise Multiple Comparison Procedures (Holm–Sidak method).

The fabric B2 with a ratio of (30/50/20 (greige cotton/bleached cotton/polypropylene) was selected as the lead. The relative effect of surface polarity on the clotting of B2 versus the most hydrophobic B9 is demonstrated in Figure 7a, which compares the R (time to fibrin formation) and K (clotting time) values in TEG experiments for a three-fiber blend (30/50/20, gc/bl/pp), B2 with a two-fiber blend (50/50, gc/pp) B9. Notably, these demonstrated similar percentage differences in the time to fibrin formation (30 percent shorter time compared with untreated controls). The K values reflected decreased clotting time by 20 and 5 percent for the more hydrophilic and hydrophobic fabrics, respectively. Thus, B2, which has pronounced hydrophilicity, was compared to B10, with a predominantly hydrophobic character (50 gc/50 pp), i.e., contact angle of 120° [4,5]. As shown in Figure 7A, the TEG-monitored clotting profile of these two nonwoven blends with different surface polarities demonstrates comparable R values, which were indicative of a 30% enhanced rate of fibrin formation compared with the untreated blood. However, a four-fold difference in clotting time (*k*) occurs when B2 and B10 are compared with untreated blood; *p* ≤ 0.05.

Based on fiber-promoted clotting and fiber/fabric properties, a lead development fabric was selected to examine fabric-promoted clotting in the Lee White clotting assay, which employs fresh blood platelets. The fabric B2 with a ratio of (30/50/20: greige cotton/bleached cotton/polypropylene) was selected as the lead. The (30/50/20: greige cotton/bleached cotton/polypropylene) was further assessed in platelet-fresh blood (Lee White clotting assay) for in vitro clotting time and compared with a sample, having equivalent percentages of each of the three fibers employed (33/33/33, gc/bl/pp). The Lee White clotting assay measures clotting as a visual assessment in time and has been employed to compare hemostatic fabrics [2]. The Lee White clotting assay assesses platelet fresh blood, enabling the in vitro assessment of the clotting effect of materials on blood platelets, which have a relatively short half-life in stored blood [39]. 

The results of the Lee White clotting assay are shown in Figure 7B. A shorter time to clot formation occurs with the more hydrophilic 30/50/20 blend, when compared with a blend having equivalent amounts of each of the three fibers (gc/bl/pp: 33/33/33). Taken together, the results shown in Figure 7A,B represent the importance of balancing hydrophilicity and hydrophobicity in the fabric to optimize clotting, and suggest the relative amounts of greige cotton/bleached cotton/polypropylene required to initiate fibrin production and surface contact activated clotting. It is also noteworthy that the 30/50/20 blend was compared between two separate nonwoven pilot processing preparations (B2 and B4-S2) and found to be comparable (Figure 7), i.e., *p* ≤ 0.05.

The final step in the development process using the triad approach employed in this study involved assessing pilot scale fabrics compared with commercially generated prototypes prepared on a manufacturer’s hydroentanglement line. The results shown in Table 4 are thrombin generation levels that were assessed to compare samples from pilot and commercial scale dressing development. The thrombin assessments were performed with whole fabric samples and the assay takes into consideration both procoagulant and anticoagulant drivers that are implicated in thrombin’s role in initiating the hydrolysis of fibrinogen to fibrin and in the downregulation of thrombin through protein C and by a tissue factor inhibitor [40]. The selected leads from both the pilot scale process and the commercially produced nonwovens process demonstrated equivalent thrombin generation at peak thrombin concentration, as judged by the velocity index. The velocity index to thrombin formation was similar for both the pilot lead and commercial prototype and it was within the expected range, as judged with a positive control formulated with a procoagulant activator. It is important to note that, in addition to velocity index, the thrombin generation profiles allow for: (a) the time of the lag phase that follows the addition of the trigger until the initiation of thrombin generation, (b) the peak thrombin generation, (c) the time to reach the peak thrombin concentration. These indicators were aligned with the velocity index and reflected the expected rate of thrombin formation. Notably, as well, that the thrombin assessment allows for the testing of larger fabric samples comparable to the Lee White clotting, which ties together the structure function results corresponding to the three vertices of the triad approach (Figure 2).

**Figure 8:**
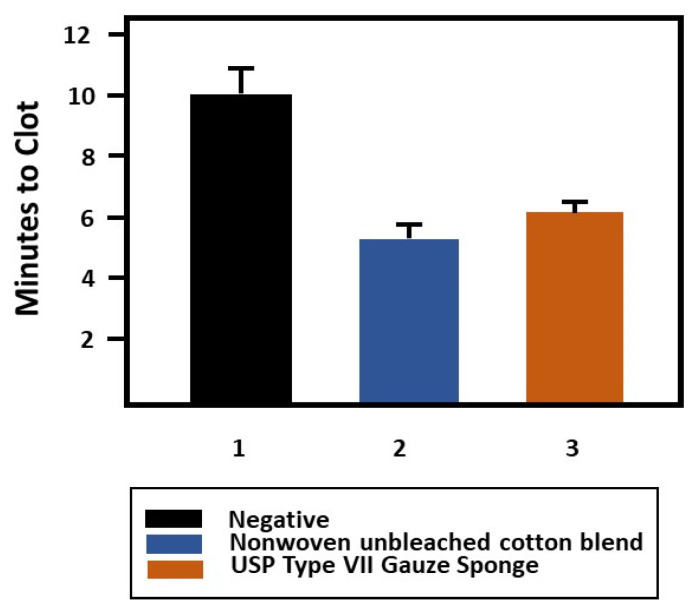
A plot of the results from the Lee White clotting assay comparing unbleached cotton-containing blend produced commercially (‘Mogul 5’, as reported in Table 4) (2) compared with USP Type VII Gauze Sponge (3). Statistical analysis done in Sigma Plot with pairwise multiple comparison procedures (Holm–Sidak method).

The demonstration of the comparable thrombin velocity index for both pilot and commercially produced fabric prototypes (*p* ≤ 0.05) prompted the assessment of the commercially produced prototype in the Lee White clotting test. Thus, the commercial prototype (Mogul 5) was compared with a commercial bleached cotton dressing gauze. As shown in Figure 8, the results of the Lee White clotting assay demonstrated a twenty percent decrease in clotting time of the greige cotton/bleached cotton/polypropylene blend, compared with a 100 percent bleached and scoured cotton gauze.

## 4. Discussion 

### 4.1. Identification of Hemostatic Greige Cotton Nonwovens

Cotton has been employed as a hemostatic dressing alternative down through the ages. The use of woven and nonwoven bleached cotton fabrics found favor in the 20th century, as wound dressings were employed for wicking and absorbance levels, providing a staple for wound treatment. However, the impetus for developing a cotton-based hemostatic dressing employing greige cotton was a finding from thromboelastography [42], where greige cotton fibers promoted shorter clotting times and a more rapid formation of fibrin than bleached cotton. Moreover, the relation of clotting initiated by greige cotton-contain fabrics correlated with surface polarity, as measured by thromboelastography and in agreement with thrombin velocity [4]. Here, we report a three-point approach to hemostatic fabric design and preparation, which connects the electrokinetically determined polar surface properties of the dressing fiber with fabric composition, in sync with absorption capacity determinations.

It is noteworthy that the employment of greige cotton in a functional skin contacting fabric is consistent with previous studies on greige cotton-containing moisture management fabrics that demonstrate fluid uptake and storage properties by way of selected fabric surface polarities suitable for hygienic products [5]. Here, we focus on the role of greige cotton-containing fabrics functioning as an absorbent hemosatic to affect the rate of fibrin and clot formation. In this regard, we have applied recently reported concepts on the mechanism of material-mediated contact activation [17,27,43]. Clotting is initiated on the fiber surface through contact activation of the coagulation cascade, and a mix of negative charge, hydrophobic and hydrophilic character initiates material surface induced thrombosis [4,27,43,44]. 

In this study, there is a trend of increasing hydrophobicity associated with shorter time to fibrin formation. In this regard, it is notable that contact angles can be used to estimate hydrophobicity. Contact angles were estimated for the lead compositions of this study, based on previous work with similar fabric compositions. Fabrics having at least 30 percent of greige cotton or 20 percent of polypropylene have contact angles from 90° to 120°, and are thus indicative of hydrophobicity [4,5]. However, hydrophilicity is also important for fluid acquisition and bleached cotton is incorporated in the blends to enhance fluid uptake and absorbency.

Combinations of the fibers utilized give rise to various degrees of swelling. However, swelling is also related to fabric porosity, void volume, and density, and more controlled studies are needed to thoroughly evaluate this approach for the effect on swelling with the selected fiber compositions. The propensity of a material to swell in a hydrated environment as in blood is associated with gelling, which has been shown to be influenced by the hydrophobic derivatization of biopolymers [32]. Several approaches to the design of hemostatic biopolymers have utilized modified chitosan and alginate in dressing motifs in this manner, showing that gelation upon contact with blood gives increased rates of hemostasis [16,45]. This is consistent with studies that have shown negatively charged surfaces initiate Factor XII activation, and is relevant to the present work as well [46].

Fabric swelling, as observed in the electrokinetic profiles of this study and others [4,5], is associated with an increased negative charge on the material’s surface. This is relevant in the context of demonstrated mechanism of action, based on surface charge. It is thought that the positively charged amino acids in the heavy chain of the Factor XII protein binds to the negatively charged surface. Upon binding to a negatively charged surface, the initiation of the conversion of Factor XII, by way of conformational changes, triggers the contact activation of serine proteases in the coagulation cascades [34]. The complex of proteins that synergize with Factor XII, including FXI, high-molecular-weight kininogen (HMWK) and pre-kallikrein, are part of the initiation pathway and participate in an amplification of the coagulation cascade on the contact surface [47]. On the other hand, studies on surface binding claim another mechanism termed an ‘adsorption-dilution’ effect from competing proteins binding to hydrophobic and hydrophilic surfaces as being responsible for contact activation [43]. However, fibrin formation through the activation of FXII is universally regarded as the material surface mechanism of contact activation [32,34,43,46]. 

It is important to note that fibrinogen, the protein that thrombin converts to fibrin, promotes cell adhesion and is the most abundant protein in blood that does so [48]. Fibrinogen binds more favorably with hydrophobic surfaces, and is influenced by physical-chemical substrate properties [41]. It has also been shown that the FXII initiated process may proceed on platelet surfaces [49]. In the mixed hydrophilic and hydrophobic surface activation study, it was postulated that contact activation leads to trace amounts of thrombin, and hydrophobic-adherent activated platelets provide binding sites for a substantial propagation of procoagulant reactions [27].

### 4.2. Absorption Capacity and Effect of Hydroentanglement Waterjet Pressure

Absorption capacity is indicative of the dressing’s capacity to retain blood and facilitate the formation of the tamponade. The nonwoven blend with the highest absorption capacity consisted of 30/50/20 (greige cotton/bleached cotton/polypropylene). As noted in the Results section, the highest amount of physiological saline was retained by the inter-fiber region of the fabrics. In this regard, the nature of water binding to cellulose inside and outside the cotton has been a subject of recent interest [50]. However, it has been shown that 14–17 separate monolayers form on the surface of cotton. Thus, the wettability of the cellulosic portion of the fiber is governed by ambient bound layers of water [51]. 

A demonstration of the importance of tuning the hydrophilic and hydrophobic fibers to optimize initiation of clotting also prompted an assessment of the effect of hydroentanglement waterjet pressure on activity. Although not shown in this work, it was observed in the course of development that increasing hydroentanglement pressure tended to increase the hemostatic activity of the fabric. This is consistent with the loosening of the waxy cuticle and exposure of more secondary cell wall cellulose. Thus, more hydrophobic and hydrophilic surface area is exposed to initiate contact activation.

### 4.3. Effect on Thrombin Release and Assessment with Lee White Clotting Assay

Both the factory-produced (Mogul 5) and pilot scale prototype (HE0249-R4) fabrics that were selected as leads demonstrated similar hyper-coagulopathic activities. An analysis of the effect of the two fabrics on thrombin generation was performed using a fluorescent thrombin substrate [40,52]. At the outset of the dressing development, it was desired to develop a fabric that would promote clotting at a rate of at least fifty percent of the rate of a procoagulant-containing dressing, i.e., a formulary containing a procoagulant as kaolin. The results of the thrombin velocity index, which represents a composite index that includes the lag phase and the time to reach peak thrombin concentration, is consistent with this relationship to procoagulant activator-containing dressing activity. Finally, employing the Lee White assay allows one to better assess the role of blood platelets. In this regard, an assessment of the factory-produced, prototype dressing in the Lee White clotting assay does show a significant increased rate of clotting when compared with a commercial one hundred percent bleached fabric. Notably, due to the limitations of blood storage, some of the assays employed in this study were limited to *n* = 2 which, to some extent, leads to higher uncertainty. 

## 5. Conclusions

This paper details the utilization of the concepts of material induced blood clotting to design, prepare and test nonwoven cotton-based fabrics for use as hemostatic dressings. The results show how greige cotton, when combined with bleached cotton and polypropylene fibers, can be employed to develop hemostatic dressings that are useful in trauma and surgical applications. Moreover, the examination of the correlation of surface polarity of the fabric to clotting activity was established, and the relation between fabric structure and fiber-based clotting profiles was demonstrated, using a variety of clotting assays that were adopted and modified to measure fabric hemostatic activity. Notably, blood clotting is a complex, and as yet, not fully understood process. Thus, applying the concepts of blood clotting to material design is a relatively subjective process. We have applied concepts and theories of material contact activation a priori to the use of specific fibers of choice. However, the design approach should also be applicable to other types of multiple fiber fabrics, where there is an interest to improve or develop new dressings with hemostatic activity.

## Figures and Tables

**Table 1:** Clotting activity of bleached 100% nonwoven cotton versus 100% greige cotton.

Sample	*R*^a^ (min)	SD ^b^	*k*^a^ (min)	SD
Bovine blood	14.6	7.8	7.3	3.2
Bleached cotton	9.6	1.6	6.6	2.2
Greige cotton	4.1	1.0	3.2	0.6

^a^*R* = time to fibrin formation and k = time to clot formation determined by thromboelastography (TEG); ^b^ SD = standard deviation, *n* = 2.

**Table 2:** List of samples of electrokinetic and time to fibrin formation data from ten compositional blends of unbleached greige cotton/bleached cotton/polypropylene [gc/bl/pp].

Sample (Density ^a^)	%Fibrin Rate (s) *	*ζ*_plateau_	∆*ζ*	*R* ^2^	*ζ*_0_	*ζ*_∞_	Swell Ratio
B1 [85gc/15bl] (41.9)	26	−31	0.14	0.902	−32.58	−29.76	1.05
B2 [30gc/50bl/20pp] (34)	32	−37	0.09	0.946	−40.62	−37.18	1.04
B3 [70gc/10bl/20pp] (37.8)	6	−35	0.09	0.97	−36.80	−33.72	1.04
B4 (60gc/2bl5/15pp] (39.8)	12	−38	0.06	0.941	−39.62	−37.17	1.03
B5 [20gc/80bl] (36.4)	2	−27	0.07	0.98	−24.94	−23.28	1.04
B6 [100bl] (27.6)	18	−25	0.09	0.99	−23.43	−21.51	1.04
B7 [100gc] (36.6)	40 **	−31	0.05	0.45	−31.61	−30.63	1.02
B8 [80bl/20pp] (30.8)	14	−41	0.05	0.881	−40.88	−38.96	1.02
B9 [50gc/50bl] (45.0)	20	−31	0.09	0.95	−32.58	−29.76	1.04
B10 [50gc/50pp] (65.0)	33	−48	0.08	0.96	−48.71	−44.94	1.04

^a^ Density is grams per meters squared. * Fibrin formation expressed as a percent difference of decreased time to fibrin formation relative to untreated blood as measured in the TEG i.e., increasing percentage = increased rate of fibrin formation. Percent differences in (*R*(Blood) − *R*(Sample))/*R*(Sample) × 100 where *R* values (time to fibrin formation) are the average of duplicate runs. R values used to compute percentages were subject to a statistical analysis prior to averaging. ** Value derived from original assessment, as shown in Table 1 for a non-fenestrated sample.

**Table 3:** Absorption Capacities of physiological saline for both intra-fiber and inter-fiber of the nonwoven blends.

Sample	SA ^a^ (mg/cm^2^)	Abs. Cap (g/g)	SD ^b^	Intra (g/g)	SD	Inter (g/g)	SD
B1 [85gc/15bl]	2.7	15.34	0.840	0.38	0.044	14.96	0.804
B2 [30gc/50bl/20pp]	3.2	17.88	1.130	0.34	0.017	17.54	1.119
B3 [70gc/10bl/20pp]	3.4	13.14	0.505	0.230	0.019	12.92	0.487
B4 (60gc/2bl5/15pp]	4.1	14.41	0.434	0.280	0.026	14.13	0.457
B5 [20gc/80bl]	3.2	14.15	0.220	0.034	0.019	13.81	0.220
B6 [100bl]	2.7	16.58	0.680	0.300	0.034	16.29	0.677
B7 [100gc] ^c^	3.26	12.95	1.526	0.38	0.055	12.57	1.534
B8 [80bl/20pp]	7.58	14.87	1.554	0.32	0.143	14.56	1.412
B9 [50gc/50bl]	3.0	15.48	0.496	0.04	0.1757	15.09	0.323
B10 [50gc/50pp]	1.76	1.97	0.424	0.13	0.025	1.84	0.403
Woven Cotton Gauze *	9.7	5.45	0.581	0.032	0.090	5.13	0.051

^a^ SA = surface area. ^b^ SD = standard deviation, *n* = 2, ANOVA, ^c^ hydroentangled nonwoven processed at 100 bar; all other samples in table are at 60 bar. * Kerlix^TM^.

**Table 4:** Results of thrombin generation assay of hydroentangled nonwoven lead prospect with controls ^a^.

Sample	Time ^1^ (min)	SD	Thrombin (nM)	SD	Time ^2^ (min)	SD	Velocity-Index	SD
Plasma control	2.0	0.0	124.5	19.9	14.5	2.1	10.2	3.3
Positive Control *	2.0	0.0	288.8	19.9	5.0	1.4	110.1	58.5
HE0249-R4	2.0	0.0	294.2	17.1	7.5	0.7	54.1	10.1
Mogul 5	2.0	0.0	294.2	30.1	8.0	0.0	49.0	5.0

^a^ Results of thrombin assessment for a final lead prepared in the pilot facility (HE0249-R4) contrasted with the commercially produced fabric (Mogul 5). * Positive control (a commercial dressing formulated with a procoagulant activator, kaolin). ^1^ Lag time is time to initial thrombin formation, ^2^ Time to peak thrombin formation. Thrombin generation assay experiments were completed with a fluorescent thrombin substrate, as previously reported [24,41]. S.D. = standard deviation, *n* = 2, Anova used for post-hoc analysis.
